# Supplementary material for: An intestinally secreted host factor promotes microsporidia invasion of C. elegans
Source: eLife. 2022 Jan 7;11:e72458. doi: 10.7554/eLife.72458 (PMC8806185; doi:10.7554/eLife.72458)
Supplement: Figure 6—figure supplement 1—source data 1. [file elife-72458-fig6-figsupp1-data1.pdf]

T14E8.4 (X-6559570-6562366)

CHROM	POS	REF	ALT	AF	allele	effect	impact	gene_name	gene_id	feature_id	transcript_biotype	nt_change	aa_change
X	6559573	A	G	0	G	synonymous_variant	LOW	T14E8.4	WBGene00043981	T14E8.4.1	protein_coding	c.1545T>C	p.Asn515Asn
X	6560363	A	G	0	G	synonymous_variant	LOW	T14E8.4	WBGene00043981	T14E8.4.1	protein_coding	c.1284T>C	p.Ser428Ser
X	6560426	A	G	0	G	synonymous_variant	LOW	T14E8.4	WBGene00043981	T14E8.4.1	protein_coding	c.1221T>C	p.Tyr407Tyr
X	6560445	T	TA	0	TA	splice_region_variant&intron_variant	LOW	T14E8.4	WBGene00043981	T14E8.4.1	protein_coding	c.1204-3_1204-2insT	NA
X	6560485	A	G	0.77	G	splice_region_variant&intron_variant	LOW	T14E8.4	WBGene00043981	T14E8.4.1	protein_coding	c.1203+7T>C	NA
X	6560524	C	G	0	G	missense_variant	MODERATE	T14E8.4	WBGene00043981	T14E8.4.1	protein_coding	c.1171G>C	p.Val391Leu
X	6560647	C	A	0.02	A	missense_variant	MODERATE	T14E8.4	WBGene00043981	T14E8.4.1	protein_coding	c.1048G>T	p.Ala350Ser
X	6560670	A	G	0	G	missense_variant&splice_region_variant	MODERATE	T14E8.4	WBGene00043981	T14E8.4.1	protein_coding	c.1025T>C	p.Val342Ala
X	6560810	T	A	0.01	A	missense_variant	MODERATE	T14E8.4	WBGene00043981	T14E8.4.1	protein_coding	c.940A>T	p.Ile314Phe
X	6560841	G	A	0.01	A	synonymous_variant	LOW	T14E8.4	WBGene00043981	T14E8.4.1	protein_coding	c.909C>T	p.Thr303Thr
X	6560869	A	T	0.02	T	missense_variant	MODERATE	T14E8.4	WBGene00043981	T14E8.4.1	protein_coding	c.881T>A	p.Val294Glu
X	6560873	C	T	0.01	T	missense_variant	MODERATE	T14E8.4	WBGene00043981	T14E8.4.1	protein_coding	c.877G>A	p.Val293Ile
X	6560876	T	C	0	C	missense_variant	MODERATE	T14E8.4	WBGene00043981	T14E8.4.1	protein_coding	c.874A>G	p.Lys292Glu
X	6560955	C	T	0	T	synonymous_variant	LOW	T14E8.4	WBGene00043981	T14E8.4.1	protein_coding	c.795G>A	p.Ser265Ser
X	6561004	C	T	0.09	T	missense_variant	MODERATE	T14E8.4	WBGene00043981	T14E8.4.1	protein_coding	c.746G>A	p.Arg249Lys
X	6561012	G	T	0.07	T	synonymous_variant	LOW	T14E8.4	WBGene00043981	T14E8.4.1	protein_coding	c.738C>A	p.Ile246Ile
X	6561013	A	T	0.01	T	missense_variant	MODERATE	T14E8.4	WBGene00043981	T14E8.4.1	protein_coding	c.737T>A	p.Ile246Asn
X	6561030	C	T	0.54	T	synonymous_variant	LOW	T14E8.4	WBGene00043981	T14E8.4.1	protein_coding	c.720G>A	p.Thr240Thr
X	6561062	C	T	0	T	missense_variant	MODERATE	T14E8.4	WBGene00043981	T14E8.4.1	protein_coding	c.688G>A	p.Ala230Thr
X	6561122	T	A	0	A	missense_variant	MODERATE	T14E8.4	WBGene00043981	T14E8.4.1	protein_coding	c.628A>T	p.Ile210Phe
X	6561199	G	T	0.03	T	missense_variant	MODERATE	T14E8.4	WBGene00043981	T14E8.4.1	protein_coding	c.551C>A	p.Thr184Asn
X	6561201	T	A	0.02	A	missense_variant	MODERATE	T14E8.4	WBGene00043981	T14E8.4.1	protein_coding	c.549A>T	p.Leu183Phe
X	6561212	A	G	0	G	splice_region_variant&intron_variant	LOW	T14E8.4	WBGene00043981	T14E8.4.1	protein_coding	c.544-6T>C	NA
X	6561830	G	A	0	A	synonymous_variant	LOW	T14E8.4	WBGene00043981	T14E8.4.1	protein_coding	c.403C>T	p.Leu135Leu
X	6562016	G	C	0.01	C	missense_variant	MODERATE	T14E8.4	WBGene00043981	T14E8.4.1	protein_coding	c.217C>G	p.Gln73Glu
X	6562145	A	T	0	T	missense_variant	MODERATE	T14E8.4	WBGene00043981	T14E8.4.1	protein_coding	c.138T>A	p.Asn46Lys
X	6562349	A	T	0	T	missense_variant	MODERATE	T14E8.4	WBGene00043981	T14E8.4.1	protein_coding	c.18T>A	p.Phe6Leu
X	6562355	T	A	0.05	A	missense_variant	MODERATE	T14E8.4	WBGene00043981	T14E8.4.1	protein_coding	c.12A>T	p.Leu4Phe
X	6562361	C	G	0.01	G	missense_variant	MODERATE	T14E8.4	WBGene00043981	T14E8.4.1	protein_coding	c.6G>C	p.Arg2Ser
